# Socioeconomic inequality in rehabilitation service utilization for schizophrenia in China: Findings from a 7-year nationwide longitudinal study

**DOI:** 10.3389/fpsyt.2022.914245

**Published:** 2022-08-26

**Authors:** Ruoxi Ding, Ping He, Xiaoying Zheng

**Affiliations:** ^1^China Center for Health and Development Studies, Peking University, Beijing, China; ^2^Asia Pacific Economic Cooperation Health Sciences Academy, Peking University, Beijing, China

**Keywords:** schizophrenia, pharmacological treatment, psychotherapy, socioeconomic inequality, China

## Abstract

**Aims:**

Few studies have focused on the utilization of rehabilitation services among people with schizophrenia. In this study, we aimed to examine the trend of pharmacological and psychotherapy service utilization among adults with schizophrenia and to identify the associated socioeconomic factors.

**Methods:**

Data were obtained from the Second National Sample Survey on Disability in 2006 and from the follow-up investigation in 2007–2013. Individuals with schizophrenia were ascertained by the combination of self-reports and on-site diagnosis by psychiatrists. Random effect logistic regression models were applied to examine the socioeconomic disparity in service utilization and the time trend in the association.

**Results:**

Overall, the percentage of individuals using pharmacological treatment services increased from 23.7 in 2007 to 55.0% in 2013, and the percentage of individuals using psychotherapy services increased from 11.4 to 39.4%. Living in rural areas, being illiterate, living in families with lower income and being uninsured were less likely to receive pharmacological treatment and psychotherapy. The pace of growth in service utilization was higher among individuals with rural residence, illiteracy or low-income status than among their counterparts with advantaged backgrounds.

**Conclusions:**

This study demonstrated an upward trend in pharmacological treatment and psychotherapy service utilization and a downward trend in socioeconomic disparity among Chinese adults with schizophrenia. Future studies to explore the reasons for the observed changes and to identify policies for improving the health service access of this vulnerable group are warranted.

## Introduction

Schizophrenia is a complex and severe psychiatric disorder with heterogeneous behavioral symptoms that generally begin in late adolescence or early adulthood (1). The onset of schizophrenia often involves disturbance of thinking, perception, language, emotion and social behaviors, and it is associated with general decay in function, social withdrawal, cognitive impairment, increased risk of suicide and reduced life expectancy (2). According to the estimation of the World Health Organization, schizophrenia affects more than 29 million people worldwide, of which 68% are from developing countries (3). The lifetime prevalence rate of schizophrenia in China was 0.83% in 2010, and it has more than doubled during the last two decades (4). As the most disabling mental illness, schizophrenia requires substantial care and a disproportionate share of mental health services. Because of the absence of a well-developed community-based care system, the caregiver burden of schizophrenia in China was possibly greater than that in most developed countries (5), imposing a considerable challenge to family, society and public health.

Most patients with schizophrenia experience chronic debilitating symptoms with frequent relapses during the progression of the disease and require long-term medical treatment and care services (6). Antipsychotic medications are currently the most effective treatment for the amelioration of symptoms and the management of acute psychotic exacerbations, and adherence is crucial to prevent relapse (7). In the meantime, patients with first-episode schizophrenia have been found to benefit from psychological therapy, such as cognitive–behavioral therapy, which was demonstrated by studies from both China (8) and developed countries (9) to be an effective treatment to reduce symptoms and enhance insight and social functioning that is not amenable to medication. It has been suggested that the combination of pharmacological treatment and psychosocial therapy increases the possibility of recovery and social participation, as well as the improvement in the quality of life, providing more efficient and comprehensive interventions for this population (10).

Socioeconomic disparities are associated with medical and mental health service utilization in various settings. Some studies have reported reduced uses of health services among low socioeconomic groups of populations due to the cost of care and the different perceptions of need (11). People with schizophrenia are diverse and vulnerable groups from multiple perspectives: geographic location, education, income, and insurance coverage, which are linked with the availability, accessibility, and affordability of medical and psychological treatment services (12). Therefore, understanding the disparities in service utilization across different socioeconomic groups is essential to identify and target potential modifiable issues for rational health system design and achieve a more equal health resource distribution for patients with schizophrenia.

Few studies have focused on the utilization of pharmacological treatment and psychotherapy among people with schizophrenia. The limited investigations were mainly conducted in Western countries. One study reported that in 1990, more than 70% of the patients diagnosed with schizophrenia in China never received any formal treatment (13). However, medical insurance coverage has made great progress since the implementation of health system reform in 2009, and community rehabilitation service infrastructure and facilities have been implemented since the 11th Strategic Plan on Chinese Disabled Persons. Nevertheless, updated reports on rehabilitation service utilization among Chinese patients with schizophrenia are still lacking. In this study, we aimed to examine the trend of pharmacological treatment and psychotherapy service utilization among adults with schizophrenia and to identify the disparities in rehabilitation service utilization and its trend between socioeconomic groups with different educational background, income and health insurance scheme characteristics in China based on nationally representative longitudinal datasets. The results of this study will provide implications for governmental policies and health care plans to meet the needs of patients with schizophrenia in China.

## Methods

### Data and participants

Data were obtained from the Second National Sample Survey on Disability in 2006 and from the follow-up investigation in 2007–2013. The aim of the 2006 survey was to investigate the prevalence, causes and severity of disabilities, as well as the sociodemographic characteristics and health service needs of the disabled. Multistage stratified random cluster sampling with probability proportional to size was employed in 734 counties (districts) 2,980 towns (streets) and 5,964 communities (villages) in all 31 provinces, autonomous regions and municipalities of China (14). The sample size of the survey was 2 526 145 individuals, representing 1.9 per 1000 non-institutionalized inhabitants.

**Figure 1:**
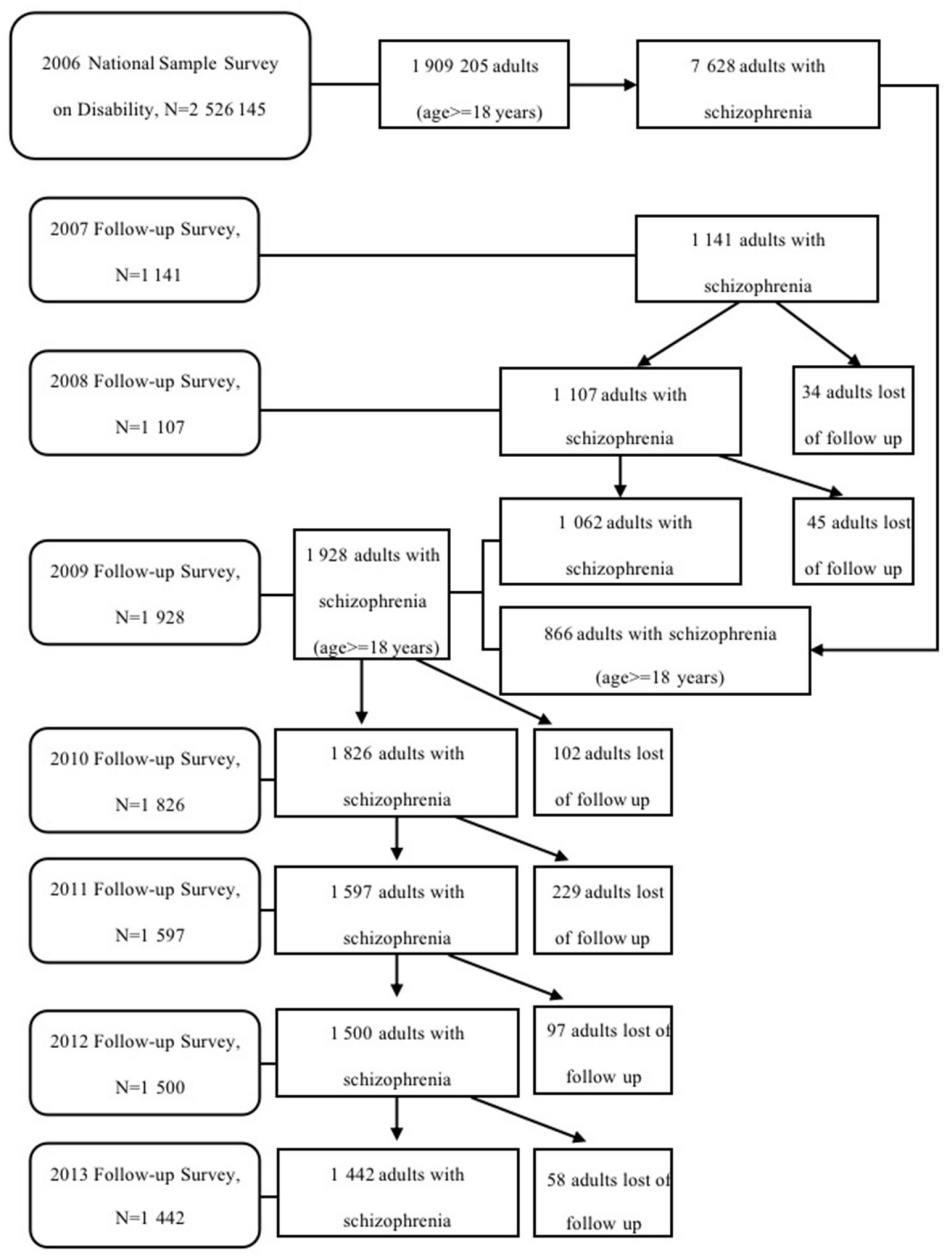
Flow chart of the study sample.

To monitor the health service utilization and the sociodemographic environment of the disabled population, a subsample of subjects with disabilities has been selected for an annual follow-up survey since 2007 (15). In the follow-up surveys, the head of the household or the main carer of the disabled people (since people with hearing, speech, intellectual or mental disability may have difficulty answering the question) were asked to complete a questionnaire at their home. In 2007 and 2008, the survey samples included individuals with disability from 734 randomly selected sites from 734 counties, with one site for each county. In 2009, an additional site from each county was added to reduce the impact of sample attrition and aging, yielding a total of 1,468 sites in 734 counties. As shown in Figure 1, of 7,628 adults with schizophrenia in the 2006 survey 1,141 were randomly selected in 2007, and 1107 and 1,062 adults were followed up in 2008 and 2009, respectively. In 2009, 866 additional cases were added, resulting in a sample of 1 928 adults with schizophrenia, of which 1 826, 1 597, 1 500 and 1 442 cases were followed up from 2010 to 2013, respectively. Overall, 94.20% of the participants were successfully followed up.

### Schizophrenia assessment

In the 2006 survey, individuals with schizophrenia were ascertained by the combination of self-reports or family members' reports and on-site diagnosis by psychiatrists based on the World Health Organization International Classification of Functioning, Disability and Health (WHO-ICF). Trained staff conducted face-to-face household interviews to identify respondents who were likely to be psychiatrically disabled. The questionnaire used in the interview was designed according to the “Guide-lines and Pinciples for the Development of Disability Statistics,” which was recommended by the United Nations (16) and has been shown to have good validity (17). If the individuals were found to have a tendency to be psychiatrically disabled, then they were referred to psychiatrists with 5 or more years of clinical experience for professional diagnosis based on The World Health Organization Disability Assessment Schedule, Version II (WHO DAS II) (18), and the International Statistical Classification of Diseases, Tenth Revision (ICD-10). The ICD-10 diagnostic criteria have been widely used in the diagnosis of schizophrenia in the Chinese population and have shown good validity and reliability (19, 20).

### Measures

The outcome variables were extracted from the information on whether individuals received pharmacological treatment/psychotherapy because of schizophrenia in the past twelve months in the survey. If the individual or their family members reported that the person with schizophrenia had received pharmacological treatment, psychotherapy or both, we considered that they had used the above services. Three outcome variables were defined: (a) pharmacological treatment as yes or no, (b) psychotherapy as yes or no; and (c) combined services (use both pharmacological treatment and psychotherapy in the same year) as yes or no.

The primary independent measurement was the survey year, which was defined as a continuous variable to examine the time trend in service utilization among people with schizophrenia. Following previous studies (21–24), the secondary independent variables included region (urban or rural area), education (illiterate or primary school and above), annual family income per capita (below or above national average) and health insurance [covered and not covered by Urban Residents' Basic Health Insurance (URBMI), Urban Employee's Basic Health Insurance (UEBMI) or New Rural Cooperative Medical Scheme (NRCMS)]. The coverage of the above universal health insurance may have a direct impact on the affordability of rehabilitation services because pharmacological treatment for schizophrenia was covered for people with URBMI, UEBMI or NRCMS. Controlled sociodemographic variables included the age of participants, sex (male or female), ethnicity (Han or minority) and marital status (married or single).

### Ethical approval

The 2006 survey and the follow-up surveys were conducted in all provinces by the Leading Group of the National Sample Survey on Disability and the National Bureau of Statistics. All the above surveys were approved by the China State Council (No. 20051104) and implemented within the legal framework governed by the Statistical Law of China (1996 amendment). Informed consent that covered participation in the survey and the clinical assessment process was signed by all the respondents. The data were deidentified, so further review of the data analysis was exempted.

### Statistical analysis

We employed descriptive statistics and Wald tests to determine time trends in sociodemographic characteristics of participants and to provide service utilization rates by socioeconomic variables in each of the surveys from 2007 to 2013. Random effect logistic regression models were applied to estimate the association between the outcome variables and independent variables (model 1), and the interaction terms between the survey time and related factors were included to examine the time trend in the association between socioeconomic status and outcome variables (model 2-model 5). A *P*-value < 0.05 was considered statistically significant. The software Stata version 14 for Windows was used for statistical analysis.

## Results

### Characteristics of participants

Table 1 shows the characteristics of the sample for each wave. From 2007 to 2013, the average age of the sample increased from 47.1 to 52.0 years (*P* trend < 0.001), and the proportion of older adults (≥65 years) increased from 13.8 to 18.4%. The proportion of rural residents first increased from 75.5 in 2007 to 77.6% in 2009 and then declined to 75.4% in 2013 (*P* trend < 0.001). The percentage of participants uncovered by health insurance dropped dramatically from 33.7 to 4.5% (*P* trend < 0.001). There was no significant difference in the distribution of sex, ethnicity, marital status, family income or education.

**Table 1:** Characteristics of participants in the surveys, 2007–2013.

**Characteristics**	**2007**	**2008**	**2009**	**2010**	**2011**	**2012**	**2013**	***P* trend**
	**(*n =* 1141)**	**(*n =* 1107)**	**(*n =* 1928)**	**(*n =* 1826)**	**(*n =* 1597)**	**(*n =* 1500)**	**(*n =* 1442)**	
Age (years), mean (SD)	47.1 (14.4)	47.8 (14.4)	48.0 (14.0)	48.8 (13.9)	50.4 (13.7)	51.3 (13.5)	52.0 (13.3)	<0.001
Older adults, *n (%)*	157 (13.8)	154 (13.9)	257 (13.3)	249 (13.6)	250 (15.7)	253 (16.9)	265 (18.4)	<0.001
Women, *n (%)*	637 (55.8)	616 (55.6)	1068 (55.4)	1012 (55.4)	882 (55.1)	827 (55.3)	795 (55.1)	0.703
Minority, *n (%)*	116 (10.2)	114 (10.3)	174 (9.0)	165 (9.0)	154 (9.6)	147 (9.8)	146 (10.1)	0.054
Single, *n (%)*	527 (46.2)	508 (45.9)	883 (45.8)	835 (45.7)	716 (44.8)	664 (44.3)	614 (42.6)	0.075
Rural Residents, *n (%)*	862 (75.5)	844 (76.2)	1496 (77.6)	1411 (77.3)	1221 (76.5)	1126 (75.1)	1087 (75.4)	<0.001
Illiterate, *n (%)*	340 (29.8)	331 (29.9)	599 (31.1)	586 (32.1)	448 (28.1)	402 (26.8)	394 (27.3)	0.114
Income below average, *n (%)*	594 (52.1)	579 (52.3)	1021 (53.0)	962 (52.7)	862 (54.0)	820 (54.7)	772 (53.5)	0.067
Uninsured, *n (%)*	385 (33.7)	257 (23.2)	346 (17.9)	244 (13.4)	99 (6.2)	99 (6.6)	65 (4.5)	<0.001

### Utilization rates of pharmacological treatment and psychotherapy services by socioeconomic characteristics

Table 2 presents the utilization rates of pharmacological treatment and psychotherapy services by socioeconomic characteristics among patients with schizophrenia. Overall, the percentage of individuals using pharmacological treatment services increased from 23.7 in 2007 to 55.0% in 2013, with a crude annual growth rate of 42.3% (95% CI: 39.7%, 45.0%). The increasing trend of the pharmacological treatment utilization rate was significant in all socioeconomic groups. The percentage of individuals using psychotherapy services increased from 11.4 in 2007 to 39.4% in 2013, with a crude annual growth rate of 45.1% (41.8%, 48.5%). The increasing trend of psychotherapy service utilization was also significant in all socioeconomic groups. A similar pattern was also observed in the utilization of combined service (using both pharmacological treatment and psychotherapy services in the same year), and the percentage increased from 6.6 in 2007 to 29.3% in 2013.

**Table 2:** Utilization rates of rehabilitation services among Chinese adults with schizophrenia, by socioeconomic characteristics, 2007–2013.

**Socioeconomic characteristics**	**2007**	**2008**	**2009**	**2010**	**2011**	**2012**	**2013**	**Annual percentage change and 95% CI**
	**(*n =* 1141)**	**(*n =* 1107)**	**(*n =* 1928)**	**(*n =* 1826)**	**(*n =* 1597)**	**(*n =* 1500)**	**(*n =* 1442)**	
**Pharmacological treatment**								
**Total**, ***n*** **(%)**	270 (23.7)	333 (30.1)	582 (30.2)	756 (41.4)	580 (36.3)	736 (49.1)	793 (55.0)	42.3 (39.7, 45.0)
**Region**, ***n*** **(%)**								
Urban	81 (29.0)	116 (44.1)	169 (39.1)	196 (47.2)	157 (41.8)	207 (55.3)	224 (63.1)	46.5 (41.3, 51.9)
Rural	189 (21.9)	217 (25.7)	413 (27.6)	560 (39.7)	423 (34.6)	529 (47.0)	569 (52.3)	41.4 (38.3, 44.6)
**Education**, ***n*** **(%)**								
Primary school and above	223 (27.8)	285 (36.7)	476 (35.8)	585 (47.2)	443 (38.6)	570 (51.9)	612 (58.4)	40.7 (37.7, 43.8)
Illiterate	47 (13.8)	48 (14.5)	106 (17.7)	171 (29.2)	137 (30.6)	166 (41.3)	181 (45.9)	49.8 (43.9, 56.2)
**Income**, ***n*** **(%)**								
≥average	170 (31.1)	209 (39.6)	355 (39.1)	411 (47.6)	314 (42.7)	385 (56.6)	413 (61.6)	43.7 (40.0, 47.6)
< average	100 (16.8)	124 (21.4)	227 (22.2)	345 (35.9)	266 (30.9)	351 (42.8)	380 (49.2)	43.9 (39.9, 48.0)
**Insurance**								
Insured	187 (24.7)	262 (30.8)	523 (33.1)	702 (44.4)	550 (36.7)	695 (49.6)	753 (54.7)	31.3 (28.5, 34.2)
Uninsured	83 (21.6)	71 (27.6)	59 (17.1)	54 (22.1)	30 (30.3)	41 (41.4)	40 (61.5)	65.3 (56.6, 74.5)
**Psychotherapy**								
**Total**, ***n*** **(%)**	130 (11.4)	176 (15.9)	287 (14.9)	380 (20.8)	379 (23.7)	496 (33.1)	568 (39.4)	45.1 (41.8, 48.5)
**Education**, ***n*** **(%)**								
Primary school and above	108 (13.5)	142 (18.3)	240 (18.1)	289 (23.3)	295 (25.7)	388 (35.3)	440 (42.0)	43.3 (39.6, 47.1)
Illiterate	22 (6.5)	34 (10.3)	47 (7.8)	91 (15.5)	84 (18.8)	108 (26.9)	128 (32.5)	51.9 (44.3, 59.8)
**Income**, ***n*** **(%)**								
≥average	79 (14.4)	106 (20.1)	178 (19.6)	217 (25.1)	206 (28.0)	251 (36.9)	292 (43.6)	43.7 (39.4, 48.3)
< average	51 (8.6)	70 (12.1)	109 (10.7)	163 (16.9)	173 (20.1)	245 (29.9)	276 (35.8)	48.7 (43.6, 54.0)
**Region**, ***n*** **(%)**								
Urban	47 (16.8)	64 (24.3)	106 (24.5)	113 (27.2)	126 (33.5)	119 (31.8)	147 (41.4)	38.7 (33.3, 44.3)
Rural	83 (9.6)	112 (13.3)	181 (12.1)	267 (18.9)	253 (20.7)	377 (33.5)	421 (38.7)	50.0 (44.8, 53.3)
**Insurance**								
Insured	92 (12.2)	144 (16.9)	258 (16.3)	349 (22.1)	359 (24.0)	475 (33.9)	551 (40.0)	37.0 (33.5, 40.7)
Uninsured	38 (9.9)	32 (12.5)	29 (8.4)	31 (12.7)	20 (20.2)	21 (21.2)	17 (26.2)	56.7 (46.5, 67.6)
**Combined services** ^**a**^								
**Total**, ***n*** **(%)**	75 (6.6)	107 (9.7)	196 (10.2)	270 (14.8)	219 (13.7)	388 (25.9)	422 (29.3)	46.5 (42.6, 50.6)
**Region**, ***n*** **(%)**								
Urban	24 (8.6)	37 (14.1)	73 (16.9)	76 (18.3)	79 (21.0)	94 (25.1)	111 (31.3)	41.0 (34.6, 47.9)
Rural	51 (5.9)	70 (8.3)	123 (8.2)	194 (13.8)	140 (11.5)	294 (26.1)	311 (28.6)	49.6 (44.7, 54.7)
**Education**, ***n*** **(%)**								
Primary school and above	65 (8.1)	87 (11.2)	167 (12.6)	207 (16.7)	171 (14.9)	299 (27.2)	331 (31.6)	43.9 (39.6, 48.3)
Illiterate	10 (2.9)	20 (6.0)	29 (4.8)	63 (10.8)	48 (10.7)	89 (22.1)	91 (23.1)	56.5 (47.1, 66.5)
**Income**, ***n*** **(%)**								
≥average	47 (8.6)	66 (12.5)	129 (14.2)	154 (17.8)	136 (18.5)	201 (29.6)	219 (32.7)	44.2 (39.2, 49.4)
< average	28 (4.7)	41 (7.1)	67 (6.6)	116 (12.1)	83 (9.6)	187 (22.8)	203 (26.3)	51.8 (45.5, 58.5)
**Insurance**								
Insured	54 (7.1)	85 (10.0)	178 (11.2)	248 (15.7)	205 (13.7)	374 (26.7)	409 (29.7)	39.2 (34.9, 43.5)
Uninsured	21 (5.5)	22 (8.6)	18 (5.2)	22 (9.0)	15 (14.1)	14 (14.1)	13 (20.0)	58.2 (46.0, 71.4)

^a^Combined services: use both pharmacological treatment and psychotherapy at the same year.

### Association between time, socioeconomic characteristics and pharmacological treatment and psychotherapy service utilization

Tables 3, 4 display the random effect model of the effect of time and socioeconomic characteristics on the utilization of pharmacological treatment and psychotherapy services. In model 1, after adjusting for sociodemographic variables, the odds of using pharmacological treatment and psychotherapy increased with time, and the odds ratios were 1.35 (95% CI: 1.31, 1.39) and 1.46 (1.41, 1.51), respectively. Living in rural areas (OR: 0.59, 95% CI: 0.49, 0.72) [0.54 (0.43, 0.67)], being illiterate [0.64 (0.55, 0.76)] [0.73 (0.60, 0.89)], living in families with lower income [0.63 (0.56, 0.72)] [0.75 (0.65, 0.88)] and being uninsured [0.55 (0.46, 0.66)] (0.54 (0.43, 0.68) were less likely to receive pharmacological treatment and psychotherapy.

**Table 3:** Random effect model for pharmacological treatment of adults with schizophrenia: odds ratio and 95% CI.

**Variables**	**Model 1^a^**	**Model 2^a^**	**Model 3^a^**	**Model 4^a^**	**Model 5^a^**
Year	1.35 (1.31, 1.39)***	1.28 (1.21, 1.36)***	1.30 (1.26, 1.34)***	1.27 (1.22, 1.33)***	1.25 (1.31, 1.39)***
Rural residents (reference = urban)	0.59 (0.49, 0.72)***	0.47 (0.35, 0.63)***	0.61 (0.50, 0.73)***	0.60 (0.49, 0.72)***	0.59 (0.49, 0.72)***
Rural residents*year		1.07 (1.00, 1.14)*			
Illiterate (reference = primary school or above)	0.64 (0.55, 0.76)***	0.65 (0.55, 0.76)***	0.38 (0.28, 0.50)***	0.64 (0.55,0.75)***	0.64 (0.55, 0.76)***
Illiterate*year			1.17 (1.10, 1.26)***		
Income < average (reference ≥ average)	0.63 (0.56, 0.72^)***^	0.63 (0.56, 0.72)***	0.63 (0.55, 0.72)***	0.43 (0.34, 0.54)***	0.63 (0.56, 0.72)***
< average*year				1.12 (1.06, 1.19)***	
Uninsured (reference = insured)	0.55 (0.46, 0.66)***	0.53 (0.44, 0.64)***	0.55 (0.46, 0.67)***	0.54 (0.45, 0.65)***	0.55 (0.41, 0.72)***
Uninsured*year					1.00 (0.91, 1.10)

^***^P <0.001, ^*^P < 0.05.

^a^All models were adjusted for age, survey year, sex, ethnicity and marital status.

**Table 4:** Random effect model for psychotherapy of adults with schizophrenia: odds ratio and 95% CI.

**Variables**	**Model 1^a^**	**Model 2^a^**	**Model 3^a^**	**Model 4^a^**	**Model 5^a^**
Year	1.46 (1.41, 1.51)***	1.26 (1.18, 1.35)***	1.43 (1.38, 1.49)***	1.38 (1.32, 1.45)***	1.47 (1.42, 1.53)***
Rural residents (reference = urban)	0.54 (0.43, 0.67)***	0.26 (0.19, 0.38)***	0.54 (0.43, 0.68)***	0.54 (0.43, 0.68)***	0.53 (0.43,0.67)***
Rural residents*year		1.22 (1.13, 1.31)***			
Illiterate (reference = primary school or above)	0.73 (0.60, 0.89)***	0.74 (0.61, 0.90)**	0.54 (0.38, 0.77)***	0.73 (0.60, 0.88)***	0.73 (0.60, 0.88)***
Illiterate*year			1.09 (1.00, 1.18)*		
Income below average (reference = above average)	0.75 (0.65, 0.88)***	0.76 (0.65, 0.88)***	0.75 (0.65, 0.87)***	0.50 (0.37, 0.66)***	0.75 (0.65, 0.88)***
Income below average*year				1.12 (1.05, 1.20)***	
Uninsured (reference = insured)	0.54 (0.43, 0.68)***	0.49 (0.87, 0.62)***	0.54 (0.43, 0.68)***	0.53 (0.42, 0.66)***	0.66 (0.46, 0.95)*
Uninsured*year					0.92 (0.82, 1.03)

^***^P < 0.001, ^**^P < 0.01, ^*^P < 0.05.

^a^All models were adjusted for age, survey year, sex, ethnicity and marital status.

Model 2–Model 5 included the interaction terms between the survey time and each type of socioeconomic characteristic to show the time trends in the association between socioeconomic status and service utilization. The odds of using pharmacological treatment among rural residents increased at a higher rate than among their urban counterparts (OR: 1.07, 95% CI: 1.00, 1.14). A similar pattern was also observed for illiterate individuals [1.17 (1.10, 1.26)] and patients from low-income households [1.12 (1.06, 1.19)]. For psychotherapy service use, the odds among rural residents increased at a higher rate than those among urban residents (OR: 1.10, 95% CI: 1.02, 1.19). A similar pattern was also observed for illiterate individuals [1.09 (1.00, 1.18)] and those from low-income households [1.12(1.05, 1.20)]. Nevertheless, the interaction terms between survey time and health insurance coverage showed no significance in either pharmacological treatment or psychotherapy.

## Discussion

This study investigated the trend of service utilization and its association with socioeconomic characteristics among people with schizophrenia in China based on a nationwide follow-up survey. We found that the average annual utilization rates of pharmacological treatment, psychotherapy and combined service were 31.2, 18.6 and 15.7%, respectively. This was lower than the result from a survey on 50 countries between 2005 and 2010, which suggested that the treated prevalence was 59% among patients with schizophrenia in Hunan Province of China (25). The national-level data used in this study make our results more representative of the issue in China. Although socioeconomic status impacts service utilization, the increasing trend was evident in all socioeconomic groups, and the gap in service utilization between advantaged and disadvantaged groups decreased. In the absence of information on resources and services for patients with schizophrenia at the global level, this study contributes to the literature on trends in access to medical treatment and psychotherapy for persons with psychiatric disorders in the eastern world.

First, we observed a mounting trend in the utilization rate of pharmacological treatment, psychotherapy and combined service for adults with schizophrenia, which increased from 23.7, 11.4, and 6.6% in 2007 to 55.0, 39.4, and 29.3% in 2013, respectively. However, a significant gap remains—a systematic review on mental health service utilization in 2004 suggested that the global median treatment rate was 67.8% (26), implying that the improvement of access to mental health services for schizophrenia in China is still far from adequate. Nevertheless, it is undeniable that China has made great improvements, with a markedly higher growth rate of service utilization for schizophrenia compared to developed countries (27). It is worth mentioning that there was a step growth in the service utilization rate between 2009 and 2010. This is probably because of the implementation of medical system reform and universal health coverage (28), as well as the severe mental illness treatment and management program at that time in China, which underlined the responsibility of the central government and local authority on the construction and promotion of a national comprehensive rehabilitation network for persons with psychosis. In addition, policy interventions may also explain another step in the growth of the service utilization rate between 2011 and 2012. The development program for people with disability in the 12th Five-year Strategic Plan, announced in 2011, was proposed to improve the rehabilitation services and infrastructure of community-based systems (29). Furthermore, from the perspective of the demand side, rising economic conditions and living standards among the Chinese population during the last decade facilitated the affordability of health services for those in need (30). Additionally, it is worth mentioning that policies or initiatives implemented after 2013 may further improve access to rehabilitation service utilization for people with schizophrenia. For example, the “Healthy China 2030” initiatives, which were issued in October 2016, claimed to comprehensively promote community rehabilitation services for mental disorders on a national scale.

Our findings suggested that socioeconomic positions affected access to service among people with schizophrenia. Illiteracy, rural residence, low income and uninsured status were associated with a reduced probability of using pharmacological treatment and psychotherapy services. Limited research has been conducted to explore the relationship between socioeconomic status and service use among this population. One study from Italy indicated a strong predictive association between social deprivation and psychiatric service uptake among schizophrenia patients (24). A similar pattern was also reported by previous research on mental health service utilization among the general population (31–33). In addition, it has been suggested that the vast majority of the rural population has little access to psychiatric medication or services since most specialized hospitals are situated in urban areas (13). Furthermore, the internalized stigma related to schizophrenia among patients with lower education, which was found by one study in China (34), may also contribute to the barriers of low socioeconomic positions to access to service. Families from low socioeconomic groups are more likely to consider external attributions for psychosis and seek informal treatment, such as herbalists, acupuncturists or shamanistic healers (35). Our findings suggested that strategies to increase access to and reduce barriers to health services among patients with schizophrenia in China and for those in developing regions should incorporate socioeconomic indicators.

There was a declining trend of socioeconomic disparities in pharmacological treatment and psychotherapy service use among adults with schizophrenia over time, with the pace of growth being higher among individuals with rural residence, illiteracy or low-income status than among their counterparts with advantaged backgrounds. In addition to the abovementioned benefits from universal health insurance coverage and rapid economic growth, the particular attention of the Chinese government and societies on vulnerable groups during the last decade may also play a part. For example, the National Continuing Management and Intervention Program for Psychoses, initiated in 2004 has been demonstrated to substantially expand the mental health service team and rebuild the hospital-community integrated service model for patients with severe psychiatric disorders (36). Meanwhile, the Disabled People Protection Law, which took effect in 2008, underlined the protection of legal rights and interests and the promotion of the well-being of the disabled population, which also includes patients with schizophrenia (30). Several assistant projects have been launched to provide subsidies and free rehabilitation services for the disabled in socially disadvantaged groups. Nevertheless, the closing gap of the service utilization rate over time was not presented in the socioeconomic characteristic of health insurance coverage, either for pharmacological treatment or for psychotherapy. The increasing rate of utilization of psychotherapy or combined services was relatively lower among uninsured patients than among their counterparts with other socioeconomic disadvantages. This probably indicates the incomprehensiveness of existing governmental interventions toward uninsured schizophrenia patients. Although China's universal health insurance scheme has gradually covered the majority of its population, it has been reported that domestic migrants and the disadvantaged population remain at the edge of the system (37). Uninsured patients with such characteristics might inevitably find it difficult to access and afford the related treatment and services for schizophrenia. Measurements such as addressing inadequate health insurance coverage and providing fee exemptions for psychiatric treatment targeting this marginalized group are urgently needed.

This study has several limitations. Caution should be taken when interpreting the results because a number of factors that may contribute to variability cannot be considered. First, the use of pharmacological treatment and psychotherapy services during the past 12 months was based on self-reported information, of which the recall errors may cause bias. Second, we are unable to perform further investigation due to the absence of information, such as supply-side factors, the number of utilizations or the detailed content of services. Third, because of the constraints of data availability, comorbidities of adults with schizophrenia, such as substance use disorder and dementia, which have been found to be associated with service utilization (38), were not allowed in this analysis. Finally, our study was based on surveys from 2007 to 2013, which are 9 years ago. Interpretation should be cautious since the service utilization rate provided in our study may not fully reflect the current situation in China.

Despite the above limitations, the main strength of our study includes a nationally representative sample drawn from multiple sites in diverse sociodemographic settings and follow-up assessment with consistent approaches and a high participation rate over a relatively long period. To the best of our knowledge, this is the first study to provide a general picture of trends and socioeconomic inequalities in related health service utilization among adults with schizophrenia in China.

## Conclusion

This study demonstrated an upward trend in pharmacological treatment and psychotherapy service utilization and a downward trend in socioeconomic disparities among Chinese adults with schizophrenia during 2007–2013. Nevertheless, the remaining gaps in the utilization rate within urban–rural residence, different educational backgrounds, income levels and insurance statuses underline the need to improve mental health service accessibility and resource allocation for socioeconomically disadvantaged populations with schizophrenia in the developing world. Future studies exploring the reasons for the observed changes and identifying policies for improving the health service access of this vulnerable group are warranted.

## Data availability statement

The data analyzed in this study is subject to the following licenses/restrictions. Data cannot be shared publicly because of legal restrictions, that is, the data contain potentially sensitive information. The State Council of China imposed the restrictions according to the Statistical Law of the People's Republic of China (1996 Amendment). A de-identified minimal dataset of the quantitative data is available upon request to researchers who meet the criteria for confidential information, by sending a request to the Data Access Committee of IPR Peking University, No. 5 Yi He Yuan Lu, Beijing 100871, China (contact via e-mail at rkyjs@pku.edu.cn). Requests to access these datasets should be directed to rkyjs@pku.edu.cn.

## Ethics statement

The 2006 survey and the follow-up surveys were conducted in all provinces of mainland China by the Leading group of the National Sample survey on Disability and the National Bureau of Statistics. And all the surveys were approved by the China State Council (No. 20051104) and implemented within the legal framework governed by the Statistical Law of China (1996 amendment). Informed consent was signed by all the respondents. We used de-identified data for analysis so the further review was exempted. The patients/participants provided their written informed consent to participate in this study.

## Author contributions

RD initiated the study, analyzed data, and wrote the original manuscript. PH provided advices on research design, data analysis, and manuscript writing. XZ originated the study, obtained the funding, supervised all aspects of its implementation, and contributed to writing the article. All authors contributed to and have approved the final manuscript.

## Conflict of interest

The authors declare that the research was conducted in the absence of any commercial or financial relationships that could be construed as a potential conflict of interest.

## Publisher's note

All claims expressed in this article are solely those of the authors and do not necessarily represent those of their affiliated organizations, or those of the publisher, the editors and the reviewers. Any product that may be evaluated in this article, or claim that may be made by its manufacturer, is not guaranteed or endorsed by the publisher.
